# The Influence of Frequency Bands and Brain Region on ECoG-Based BMI Learning Performance

**DOI:** 10.3390/s21206729

**Published:** 2021-10-11

**Authors:** Wongyu Jung, Seokbeen Lim, Youngjong Kwak, Jeongeun Sim, Jinsick Park, Dongpyo Jang

**Affiliations:** 1 Department of Electronics and Computer Engineering, Hanyang University, Seoul 04763, Korea; jyg2480@gmail.com; 2 Department of Biomedical Engineering, Hanyang University, Seoul 04763, Korea; seokblim@gmail.com (S.L.); yjmj123123@hanyang.ac.kr (Y.K.); shimje83@naver.com (J.S.); jinseek@gmail.com (J.P.)

**Keywords:** brain–machine interface, frequency band, brain area

## Abstract

Numerous brain–machine interface (BMI) studies have shown that various frequency bands (alpha, beta, and gamma bands) can be utilized in BMI experiments and modulated as neural information for machine control after several BMI learning trial sessions. In addition to frequency range as a neural feature, various areas of the brain, such as the motor cortex or parietal cortex, have been selected as BMI target brain regions. However, although the selection of target frequency and brain region appears to be crucial in obtaining optimal BMI performance, the direct comparison of BMI learning performance as it relates to various brain regions and frequency bands has not been examined in detail. In this study, ECoG-based BMI learning performances were compared using alpha, beta, and gamma bands, respectively, in a single rodent model. Brain area dependence of learning performance was also evaluated in the frontal cortex, the motor cortex, and the parietal cortex. The findings indicated that BMI learning performance was best in the case of the gamma frequency band and worst in the alpha band (one-way ANOVA, F = 4.41, *p* < 0.05). In brain area dependence experiments, better BMI learning performance appears to be shown in the primary motor cortex (one-way ANOVA, F = 4.36, *p* < 0.05). In the frontal cortex, two out of four animals failed to learn the feeding tube control even after a maximum of 10 sessions. In conclusion, the findings reported in this study suggest that the selection of target frequency and brain region should be carefully considered when planning BMI protocols and for performing optimized BMI.

## 1. Introduction

The brain–machine interface (BMI) was designed to restore sensorimotor functions in people in need of rehabilitation, as well as to control external devices using patterns of electrical signals generated by cortical neurons in the brains of people using neuroprosthetics [1,2]. The BMI technique has been widely used as a neural model for understanding mechanisms related to issues such as brain plasticity beyond the meaning of the neural prosthesis [3,4]. In general, spikes or a local field potential (LFP) from deep electrodes were used as neural information in BMI systems [5]. Chaptin et al. (1999) showed that a BMI rodent animal model could learn to spontaneously control a robot arm in order to obtain a reward using multi-channel spike signals in real-time [6]. Such invasive types of neural information acquisition methods had much better spatial resolution, and the signal was of higher quality compared to scalp EEG [7,8].

Electrocorticography (ECoG) has an advantage in that it is possible to record a signal of better quality than that of scalp EEG because electrodes are placed on the surface rather than being inserted into the brain [7,8]. Leuthardt et al. reported on possible ECoG-based features of BMI when the movement of the cursor or joystick was controlled [7]. Costecalde demonstrated that BMI could be used to control food dispensers through freely moving rodent ECoG signals and verified that the system could operate well for a long period [9]. They also reported that the gamma band in the motor cortex was used in operating the system [9] because the oscillation of the gamma band was generated in a local cortex area, and it was related to neuronal activation [10]. Thus, many researchers have continued to report findings from BMI studies with ECoG signals on the motor cortex showing that features of the gamma band have potential for use in BMI studies [11,12,13,14,15].

Analogous to motor learning, if the process of BMI learning is similar to a motor learning task [16], the animal should be able to modulate not only the gamma band of brain oscillation but also the oscillation of the alpha and beta bands for BMI learning and external device control [17,18,19]. In this regard, J.R. Wolpaw and D.J. McFarland reported results showing that a two-dimensional cursor could be made to move using the alpha band and the beta band [17]. Engelhard et al. showed that monkeys are able to spontaneously control gamma oscillation to drive a BMI system [18]. Although these studies show the applicability of various frequency bands to BMI, there are few studies demonstrating how learning efficacy, such as learning speed and capability, may differ depending on frequency band, raising the question of whether a particular frequency may be optimal. In addition to frequency band, various areas of the brain have been selected previously as targets for inserting an electrode. Traditionally, the motor cortex has been the main target, but recently, other areas, including the parietal cortex, have been targeted as the brain cortex for use in BMI applications [20]. However, although the selection of target frequency and brain region appears to be crucial to obtaining optimal BMI performance, the direct comparison of BMI learning performance as it relates to brain region and frequency band has not been examined in detail. Thus, for this report, two comparison studies were performed to validate ECoG-based BMI learning performance with respect to spectrum power band (alpha, beta, and gamma bands) and brain region (the frontal cortex, the motor cortex, and the parietal cortex).

## 2. Materials and Methods

### 2.1. Brain–Machine Interface (BMI) Learning Session: Feeding Tube Control by ECoG Signal

**Figure 1:**
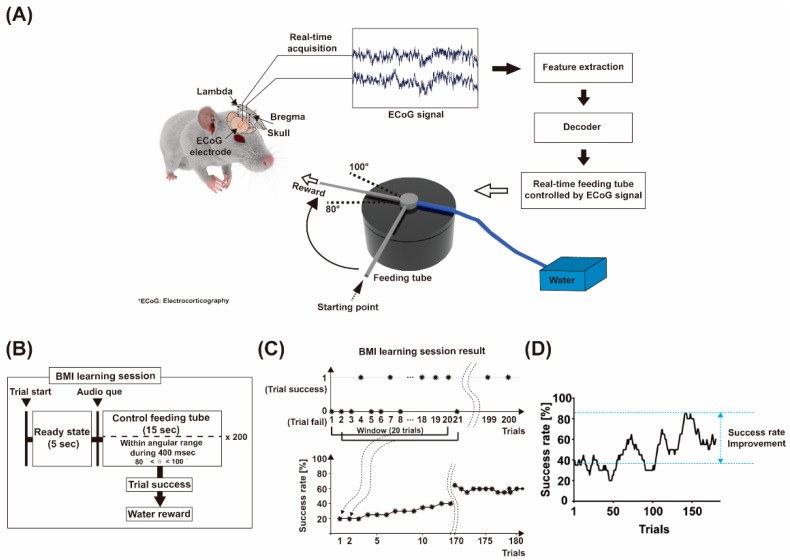
Brain–machine Interface (BMI) learning session. (**A**) ECoG-based BMI procedure. The water feeding tube was controlled by decoding the features of the ECoG signal measured in real-time. (**B**) Block diagram of the BMI learning session with 200 trials. (**C**) The calculation of success rate using the ratio of the number of success trials among 20 consecutive trials. (**D**) The success rate improvement, defined as the difference between the first success rate and maximum success rate within the session.

We conducted an ECoG-based BMI learning session by making the animal neural control of the water feeding tube. For ECoG signal acquisition, two screw-type recording electrodes were implanted on the skull. Before the BMI learning session, animals were acclimated to a custom acrylic box environment, which was designed to include a long rectangular-shaped hole to accommodate a feeding tube at one end (Figure 1A). The BMI experimental environment and the BMI protocol can be seen in the attached supplementary materials (Figure S1) and short video. During the adaptation period, a water feeding tube system rotated from the starting point at a 90° angle for the animals to acclimate to the water feeding tube over 3–4 days, as shown in Figure 1A. The animals could take in water when the feeding tube arrived at a 90° angle in the acrylic box. Each animal’s body weight was monitored daily to prevent a decrease below 90% of its initial body weight. Before the BMI learning session, the animals were deprived of water for about 12 h to encourage them to perceive water as a reward. All BMI learning experiments were conducted from 6:00 p.m. to 9:00 p.m. A single BMI learning trial consisted of a ready state (5 s) and a learning state (15 s), as shown in Figure 1B. After finishing the ready state, a 4.5 kHz beep sound alerted the animal to recognize the beginning of the learning state. In this state, the feeding tube was controlled based on their ECoG signals. The average spectrum power within a specific frequency band (i.e., a 30–55 Hz gamma band) was calculated from ECoG data. The feeding tube angular position was updated with the spectrum power at intervals of 200 ms. The subjects were required to keep a feeding tube at specific angular ranges (between 80° and 100°) for 400 ms (Figure 1A). If they succeeded in maintaining a feeding tube at those ranges, they could take in water as a reward (Figure 1A), and it was counted as a success trial. If not, within 15 s after the beep sound, the trial was recorded as a fail trial (Figure 1B,C; please refer to Figure S1 for details on failure conditions). Details are described in the section “neural control of the water feeding tube system”.

To evaluate BMI learning performance, we obtained a learning curve with the success rate calculated as the ratio of the number of success trials among 20 consecutive trials (Figure 1D). In addition, success rate improvement was defined to estimate BMI learning performance as the difference between the first success rate and maximum success rate within the session.

### 2.2. The BMI Learning Performance Comparison Protocol: Frequency and Brain Region

**Figure 2:**
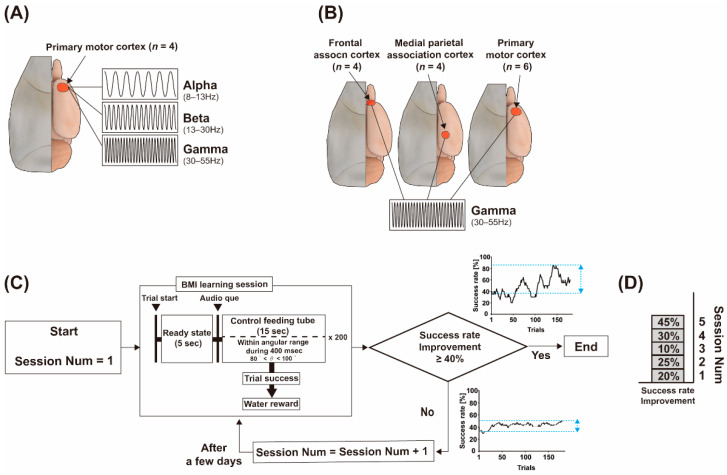
BMI learning performance comparison protocol: frequency and brain region. (**A**) Frequency-dependent BMI learning experiment with three frequency bands—Alpha: 8–12 Hz; Beta: 13–30 Hz; Gamma: 30–55 Hz. (**B**) Brain area-dependent BMI learning experiment with three brain regions: primary motor cortex, medial parietal association cortex, and frontal association cortex. (**C**) The repeated multiple BMI learning session protocol. The BMI learning session was repeated until the success rate improvement condition was satisfied. (**D**) The number of conducted sessions and success rate improvement of each session.

Two comparison studies were designed and conducted to validate ECoG-based BMI learning performance with respect to spectrum power band and brain region. In the first study, the effect of frequency band on BMI learning performance was evaluated with alpha (8–13 Hz), beta (13–30 Hz), and gamma (30–55 Hz) bands in the rat with the electrode placed on the primary motor cortex (Figure 2A). BMI learning performance for one of the frequencies was validated with repeated multiple BMI learning session protocols as shown in Figure 2C because it could be influenced by various factors such as daily conditions. First, the animal performed a BMI learning session by modulating the specific frequency power of the ECoG for feeding tube control as described in the previous section. If the success rate improvement in the BMI learning session was not greater than 40% (empirically defined criterion), the same BMI learning session was performed again a few days later (Figure 2C). The BMI learning sessions were repeated until success rate improvement was above the criterion. The maximum number of sessions was 10. Finally, we recorded the number of sessions conducted for fulfilling the 40% success rate improvement criterion (Figure 2D). After finishing this protocol with a specific frequency, the entire protocol was conducted again at the other two frequencies with the same animal. Four rats were used in this frequency comparison study.

In the second study, the influence of brain region on BMI learning performance was evaluated at the frontal cortex, the motor cortex, and the parietal cortex, as depicted in Figure 2B. Recording electrodes were implanted in one of three brain regions in the animal. BMI learning performance was validated using the repeated multiple BMI learning session protocol outlined in Figure 2C. All protocols were conducted with a gamma frequency band. A total of 14 rats took part in this study (four rats per frontal and parietal cortex, six rats to the motor cortex).

### 2.3. Surgical Procedure

Fourteen healthy, adult Sprague Dawley rats (300–350 g, male, Koatech, Pyeongtaek, 17711 Korea) were used in the ECoG-based BMI learning experiments. Before surgery, these animals were housed in 12 h light/dark cycles with lights on at 7:00 a.m. and were provided food and water ad libitum. After injection with the anesthetic zoletil (0.1 mL/100 g, 5 mg/mL, Vibrac, France), screw-type electrodes (diameter 1 mm, depth 3 mm) were implanted on the skull. We determined electrode location with reference to Paxinos and Watson’s Rat Brain Atlas [21]. Animal body temperature was maintained at 37 °C using a heating system (TCAT-2, Harvard Apparatus, Holliston, MA 01746, USA).

In the frequency-dependent BMI learning study, two recording electrodes were implanted in the bilateral primary motor cortex (AP: +2 mm, ML: ±3 mm). In the case of the brain area-dependent BMI learning experiment, the recording electrodes were implanted in the bilateral frontal association cortex (AP: +5.64 mm, ML: ±1.9 mm), the bilateral medial parietal association cortex (AP: −4.08 mm, ML: ±2.1 mm), and the bilateral primary motor cortex (AP: +2 mm, ML: ±3 mm). Reference and ground electrodes were implanted symmetrically in the nearby lambda (AP: −11 mm, ML: ±1.5 mm) using screw-type electrodes. After inserting the electrodes, a pedestal connector (P1 Technologies, Roanoke, VA, USA) was connected and fixed between the electrodes and sealed with dental cement (DentKist, Gunpo 15875, Korea). The animals were intramuscularly injected with antibiotics at the end of surgery to prevent the inflammation of surgical areas. Animals were given adequate time to recover in their cages after the surgery, consistent with the Guide for the Care and Use of Laboratory Animals. We constantly monitored the recovery state of the animals based on their weight and the inflammation state of the surgical area. After recovery, animals were typically handled for several days before the start of experimental sessions. All experimental procedures were approved by the Hanyang University Institutional Animal Care and Use Committee (IACUC No. 2019-0151A).

### 2.4. ECoG Recording

We connected a swivel cable (P1 Technologies, Roanoke, VA, USA) to the animal’s head pedestal. The swivel cable was then attached to the swivel commutator (P1 Technologies, Roanoke, VA, USA) to provide a stable brain signal when the animal moved freely. ECoG data were recorded using g.USBAMP at 600 Hz (g.TEC Medical Engineering, 4521 Schiedlberg, Austria) from different locations according to the frequency-dependent and the brain area-dependent BMI learning experiment (Figure 2A,B). We monitored real-time data using the brain signal recording software BCI 2000 (National Center for Adaptive Neurotechnologies lab, New York, NY, USA). The field trip toolbox and MATLAB (The Mathworks, Natick, MA, 01760 USA) were used on a 32-bit Windows 7 operating system for neuroprosthetic control of the water feeding tube system (Microsoft, Redmond, WA, 98052, USA).

### 2.5. Neural Control of the Water Feeding Tube System

We designed an online decoder that could update the angle of the feeding tube using the subject’s ECoG signal during BMI learning sessions. This decoder updated the angular position at intervals of 200 msec. ECoG data were recorded and saved to analyze brain patterns while the online decoder operated. The decoder algorithm used in those sessions has been described in previous studies [22,23].

θ_v_ = C × (G_1_ × Spectrum power_(right hemisphere)_ + G_2_ × Spectrum power_(left hemispere)_) (1)

In the above formula, spectrum power was calculated from ECoG data for each right and left hemisphere. ECoG data recorded at every 200 ms were bandpass filtered in the range of 0.1 Hz to 200 Hz. We also used each 60 Hz, 120 Hz, and 180 Hz notch filter to attenuate noise. The fast Fourier transform (FFT) was conducted with these data to calculate spectrum power. The target spectrum band was selected for the angular update. In the case of the frequency-dependent BMI learning experiments, as shown in Figure 2A, we used three different target frequency spectra, namely, the alpha band (8~13 Hz), the beta band (13~30 Hz), and the gamma band (30~55 Hz), to control the angular position of the feeding tube. In the case of the brain-area-dependent BMI learning experiments shown in Figure 2B, we used the gamma band as the target frequency band to control the angular position of the feeding tube.

G1 and G2 were the weight values to balance the spectrum power of the brain signal between the left and right hemispheres. To initialize the G1 and G2 values, we recorded and averaged spectrum powers for 10 min before the BMI learning sessions. The G1 and G2 values were allocated with the reciprocal of each hemisphere spectrum power and multiplied by 0.5. The sign (+ or −) was randomly allocated (‘+’: forward direction and ‘−’: backward direction). C value was the fixed constant value used to modulate BMI learning difficulty. We empirically selected the C value to have roughly a 20% success rate at the beginning of the BMI experiments.

## 3. Results

### 3.1. BMI Learning Performance Comparison Study for Frequency Bands

**Figure 3:**
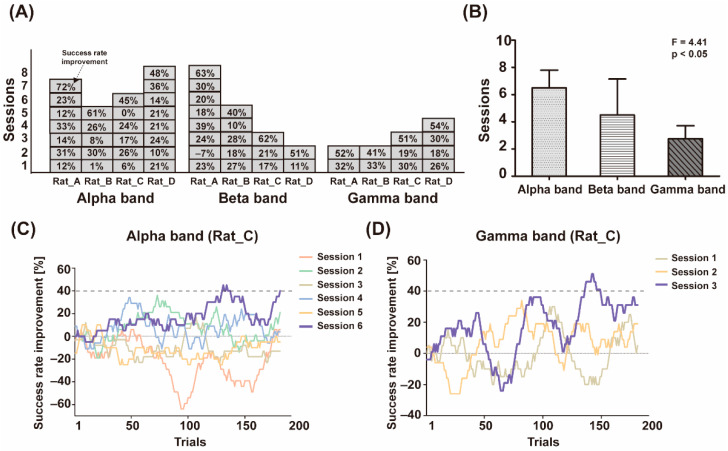
Frequency-dependent BMI learning performance comparison. (**A**) Total sessions and success rate improvements for frequency-dependent BMI learning of each animal. (**B**) Average sessions for successful frequency-dependent BMI learning. (**C**) BMI learning pattern within each session with the alpha band of Rat_C; (**D**) with the gamma band. The dashed line represented success rate improvement criterion.

When comparing the number of sessions required to achieve the success rate improvement criterion for each band, there were BMI learning performance differences among the frequency bands. As shown in Figure 3A, in the case of “Rat_A”, the animal met the criterion after seven sessions in the case of the alpha frequency band, and after eight sessions in the case of the beta band. In contrast, only two sessions were conducted for the gamma frequency band. Figure 3B shows a graph of the average number of sessions to learning success in each frequency band from the four animals. The subjects finally succeeded in BMI learning after 2.8 ± 1.0 sessions in the gamma band, 4.5 ± 2.6 sessions in the beta band, and 6.5 ± 1.3 sessions in the alpha band. The animals showed significantly faster learning performance when the gamma band was used (One-way ANOVA, F = 4.41, *p* < 0.05). Figure 3C,D shows a BMI learning pattern within each session after multiple repeated sessions.

### 3.2. BMI Learning Performance Comparison Study on Brain Region

**Figure 4:**
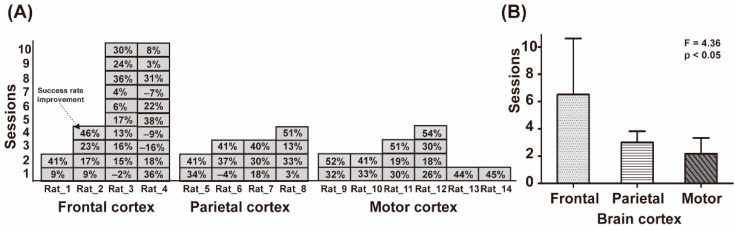
Results for brain-area-dependent BMI learning for each group. (**A**) Total sessions for brain-area-dependent BMI learning of each animal (frontal and parietal cortex group, *n* = 4; motor cortex group, *n* = 6). (**B**) Graphs of average sessions for successful brain-area-dependent BMI learning.

To assess BMI learning performance depending on brain region (the frontal cortex, the parietal cortex, and the motor cortex), four rats per frontal and parietal cortex, and six rats assigned to the motor cortex, participated in repeated BMI learning sessions (Figure 2C). As a result, the animals succeeded in BMI learning after 2.2 ± 1.2 sessions in the case of the motor cortex, 3.0 ± 0.8 sessions in the case of the parietal cortex, and 6.5 ± 4.1 sessions in the case of the frontal cortex (Figure 4B). Animals showed significantly better BMI learning performance when the motor cortex was used (one-way ANOVA, F = 4.36, *p* < 0.05). As shown in Figure 4A, in the case of the frontal cortex area, two animals (Rat_3 and Rat_4) failed to show learning performance above the success rate improvement criterion even after the maximum of 10 sessions.

## 4. Discussion

In this study, ECoG-based BMI learning performance was explored as a function of frequency band (alpha, beta, and gamma band) and brain area (frontal, motor, and parietal cortex). Each animal conducted multiple learning sessions to control a water feeding tube through a specific frequency band power of ECoG signals measured in one of three brain regions. As a result, the BMI learning performance was found to be best in the gamma frequency band. In addition, better BMI learning performance was shown in the primary motor cortex. The findings presented in this study suggest that the motor cortex and gamma frequency band represent the best target parameters for planning BMI protocols and for achieving optimized BMI.

Many previous BMI studies have used the gamma band frequency as a control feature [11,12,13,14,15] based on the fact that it is related to neuronal activation (spike or local field potential) generated by local neurons [10]. In the same line, the best BMI learning performance with the gamma band in the motor cortex shown in this study provides indirect support for the previous rationale concerning the use of the gamma band for external device control. Dubey and Ray compared the LFP and ECoG signals in the gamma band in monkeys and reported that the two signals are similar [19]. Engelhard et al. showed that monkeys voluntarily regulate the gamma band signal in a BMI system [18]. One of the interesting findings of this study was that the animals did finally succeed in BMI learning after some sessions in the case of the beta band and in the case of the alpha band, but the learning performance in both cases was lower than that for the gamma band. These findings suggested that the same animal could learn how to modulate any frequency on the motor cortex in BMI learning. Previous studies have reported that alpha or beta bands could be used to control the BMI system [24,25]. It is noteworthy that Wolpaw showed, in human subjects, a two-dimensional cursor controlled through alpha and beta band powers from a scalp-EEG signal [17]. In addition, Sugata et al. reported a significant correlation between the functional connectivity of the alpha band and BMI decoding performance, which occurs during actual movement and imagined movement [24].

Most of the BMI controlled the system through brain signals from the primary motor cortex. Leuthardt et al. reported that the signals measured in the motor cortex control the BMI system most clearly and directly reflected the user’s intention to interact with the environment using their arms, legs, and hands [25]. Andersen et al. also suggested that the motor cortex had a high correlation with movement and could provide a high degree of freedom signal-related to the move [20]. The finding in our study that better BMI performance occurred in the motor cortex rather than the parietal and frontal cortex appears to be consistent with previous reports. Since the water feeding tube to be controlled in this study also had a moving feature similar to a limb control, the ECoG signal from the motor cortex could be modulated more easily than from other areas of the brain. Nevertheless, the neural signal of the parietal cortex contained information related to the user’s intention and could be decoded to control the BMI system [26] because the existence of a functional connectivity between the motor cortex and the parietal cortex on movement has long been known. In a similar context, the animals with an electrode on the parietal cortex in our study also succeeded in BMI learning after a few sessions. In the case of BMI in which signals from the frontal cortex are used, there have been a number of studies on implementing the BMI system using brain signals obtained from the frontal cortex [27,28,29]. Leinders et al. showed that BMI training is possible in humans using dorsal lateral prefrontal cortex (DLPFC) ECoG signals [27]. Widge and Moritz also implemented a closed-loop limbic neurostimulator controlled through the BMI system using spike signals measured in the prefrontal cortex of rodents [29]. In contrast to previous studies, in this study, the learning performance for the frontal lobe cortex was lower compared to the other two areas of the brain (Figure 4A,B). Rat_3 and Rat_4 in the frontal cortex group, shown in Figure 4A, even failed to learn to control the feeding tube within the maximum number of sessions. This reflects the conclusion that the frontal regions should be carefully considered as a target brain area for BMI study.

## 5. Conclusions

In this study, we implemented a rodent ECoG BMI system and compared BMI learning performance using multiple frequency bands and brain regions. The findings indicated that BMI learning performance was best in the case of the gamma frequency band and worst in the alpha. In brain area -dependence experiments, better BMI learning performance was shown in the primary motor cortex. In the frontal cortex, two of four animals failed to show a learning performance above the success rate improvement criterion even after the maximum of 10 sessions. The findings presented in this study suggest the selection of target frequency and brain region need to be carefully considered in planning future BMI studies.

## Data Availability

Not applicable.

## Supplementary Materials

The following are available online at https://www.mdpi.com/article/10.3390/s21206729/s1, Figure S1: ECoG-based BMI experiment environment, Video S1: ECoG-based BMI experiment environment short video.
