# Supplementary material for: The Influence of Frequency Bands and Brain Region on ECoG-Based BMI Learning Performance
Source: Sensors (Basel). 2021 Oct 11;21(20):6729. doi: 10.3390/s21206729 (PMC8541475; doi:10.3390/s21206729)
Supplement: Supplementary file 1 [file sensors-21-06729-s001.zip › sensors-1344665_Supplementary materials.pdf]

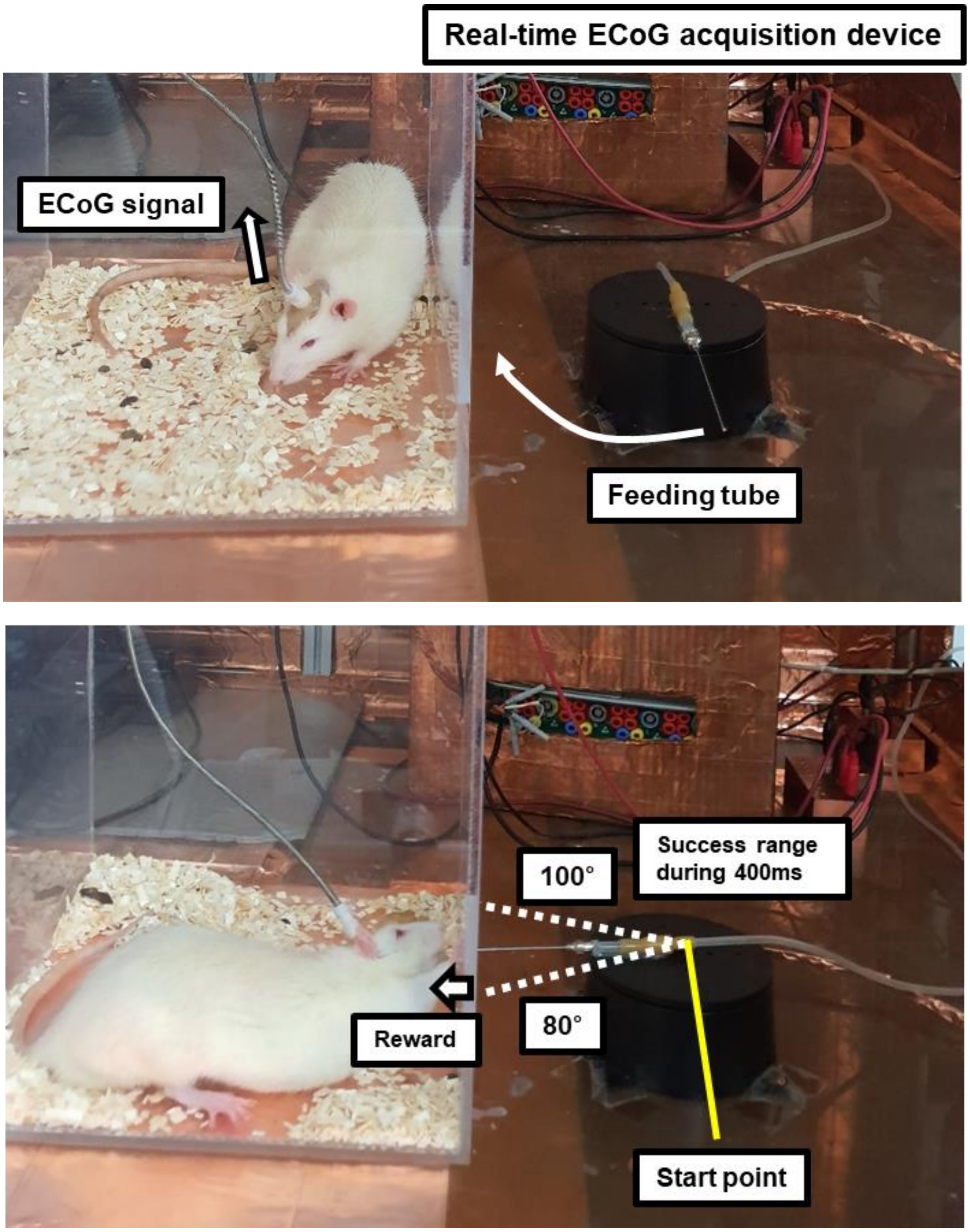

Figure S1. ECoG-based BMI experiment environment. Animals should control the water feeding tube by modulating their ECoG signals. In the BMI learning session, keeping the water feeding tube between 80 and 100 degrees from the start point for 400 ms was recognized as a trial success and animals got the water as a reward. We considered being failure-cases not to achieve the success condition. For example, in the case that animals didn't keep a tube at those ranges given by 400 msec during 15 seconds of the learning state, we decided as a trial failure. Also, in the case that the tube was reached a maximum angle (180° from the start point) of the motor system, we decided as a trial failure because we thought that animals lost their control of the system. And, in the case that the tube wasn't moved from the start point, we decided as a trial failure because we thought that animals didn't learn how to control the BMI system.
